# CCR6 Functions as a New Coreceptor for Limited Primary Human and Simian Immunodeficiency Viruses

**DOI:** 10.1371/journal.pone.0073116

**Published:** 2013-08-29

**Authors:** Salequl Islam, Nobuaki Shimizu, Sheikh Ariful Hoque, Atsushi Jinno-Oue, Atsushi Tanaka, Hiroo Hoshino

**Affiliations:** 1 Department of Virology and Preventive Medicine, Gunma University Graduate School of Medicine, Maebashi, Gunma, Japan; 2 Cell and Tissue Culture Laboratory, Centre for Advanced Research in Sciences, University of Dhaka, Dhaka, Bangladesh; 3 Research Institute for Microbial Diseases, Osaka University, Osaka, Japan; University of Pittsburgh Center for Vaccine Research, United States of America

## Abstract

More than 12 chemokine receptors (CKRs) have been identified as coreceptors for the entry of human immunodeficiency virus type 1 (HIV-1), type 2 (HIV-2), and simian immunodeficiency viruses (SIVs) into target cells. The expression of CC chemokine receptor 6 (CCR6) on Th17 cells and regulatory T cells make the host cells vulnerable to HIV/SIV infection preferentially. However, only limited information is available concerning the specific role of CCR6 in HIV/SIV infection. We examined CCR6 as a coreceptor candidate in this study using NP-2 cell line-based in-vitro studies. Normally, CD4-transduced cell line, NP-2/CD4, is strictly resistant to all HIV/SIV infection. When CCR6 was transduced there, the resultant NP-2/CD4/CCR6 cells became susceptible to HIV-1HAN2, HIV-2MIR and SIVsmE660, indicating coreceptor roles of CCR6. Viral antigens in infected cells were detected by IFA and confirmed by detection of proviral DNA. Infection-induced syncytia in NP-2/CD4/CCR6 cells were detected by Giemsa staining. Amount of virus release through CCR6 has been detected by RT assay in spent culture medium. Sequence analysis of proviral DNA showed two common amino acid substitutions in the C2 envelope region of HIV-2MIR clones propagated through NP-2/CD4/CCR6 cells. Conversely, CCR6-origin SIVsmE660 clones resulted two amino acid changes in the V1 region and one change in the C2 region. The substitutions in the C2 region for HIV-2MIR and the V1 region of SIVsmE660 may confer selection advantage for CCR6-use. Together, the results describe CCR6 as an independent coreceptor for HIV and SIV in strain-specific manner. The alteration of CCR6 uses by viruses may influence the susceptibility of CD4+ CCR6+ T-cells and dendritic cell subsets *in vivo* and therefore, is important for viral pathogenesis in establishing latent infections, trafficking, and transmission. However, clinical relevance of CCR6 as coreceptor in HIV/SIV infections should be investigated further.

## Introduction

Human and Simian Immunodeficiency viruses (HIV and SIV) gain access to susceptible cells by binding their mature envelope (env) glycoprotein, gp120 to CD4 as receptor [1] and then to a coreceptor, preferably one of two chemokine receptors (CKRs), CCR5 and CXCR4 [2]. The binding events induce activation of another viral protein, gp41, which eventually create membrane fusion on the cell surface [3]. The major coreceptors utilization by infectious viruses determines their tropism and classified HIV/SIV primarily into three groups: R5 viruses (use CCR5 alone as coreceptor), R5X4 viruses (use both CCR5 and CXCR4) and X4 viruses (use CXCR4 alone), which are less frequent. However, viruses begin their primary infections through CCR5 receptor, and change their tropism to CXCR4, probably expand to more coreceptor-usage [4].

Typical chemokine family comprises 20 receptors, assigned to four subfamilies. The majority of the CKRs, namely CCR1, CCR2b, CCR3, CCR4, CCR5, CCR8, CCR9, CXCR1, CXCR2, CXCR4, CXCR5, CXCR6 and CX3CR1 have been identified already as coreceptors used by various HIV and SIV strains [5–7]. Uses of alternative coreceptors in CCR5-depleted condition were reported recently *in vivo* in sooty mangabeys infection [8]. Therefore, viral infection using alternative coreceptor other than CCR5 exists *in vivo*. However, only limited information is available concerning the usage of CC chemokine receptor 6 (CCR6), as HIV/SIV coreceptor [9]. CCR6 was identified initially as an orphan receptor and named as STRL22 [10] and subsequently renamed. The receptor is known to express on Th17 cells, regulatory T cells and memory T cells [11–13]. One recent study shows the coexpression of CCR6 in the presence of CCR4 and CXCR3 makes T cells more permissive to HIV infection [14]. Therefore, we sought to examine extensively whether CCR6 can support the coreceptor activity independently for HIV-1, HIV-2 or SIV strains and/or isolates. We examined a panel of 11 laboratory-adapted strains and seven primary isolates of HIV/SIV for their ability to gain entry into cells expressing CCR6 in collaboration with CD4. To our knowledge, this is the only detail report to describe CCR6 as an independent coreceptor for HIV and SIV.

## Materials and Methods

### Cells

A human T-cell line, C8166 that naturally expresses CD4 and CXCR4, were transduced with CCR5 to prepare C8166/CCR5 cells and used for the preparation of viral stocks of HIV-1, HIV-2 and SIV strains [15]. A panel of established human cell lines from different organs was examined for the expression of mRNA of CCR6 and to amplify its open reading frame (ORF). T-cell lines, ATL-1K [16], C8166, Jurkat [17] and a B-cell line, Daudi [18], were cultured in RPMI 1640 medium (NISSUI Co., Inc., Tokyo, Japan) containing 10% fetal bovine serum (FBS). The human glioma cell line, NP-2 is a well-reputed cell-system tool for coreceptor examination study [15]. We used Eagle’s minimum essential medium (EMEM) (NISSUI Co., Inc.) with 10% FBS to maintain NP-2 and its derivatives expressing CD4 and/or CCR6. Hepatoma cell lines, huH1 [19] and HepG2 [20] were cultured in Dulbecco’s modified EMEM (DMEM) (NISSUI Co., Inc.) containing 10% FBS. Human peripheral blood mononuclear cells (PBMC) were isolated from blood of healthy donors by Ficoll-Paque density gradient centrifugation (Pharmacia, Uppsala, Sweden). The amphotropic packaging cell line Phoenix-Ampho [21] was maintained in DMEM containing 10% FBS.

### Viruses

Both laboratory-adapted strains and primary isolates of HIV-1, HIV-2, and SIV were utilized to check the coreceptor use of CCR6. The established cell line-adapted dual tropic HIV-1 strains GUN-1WT, GUN-1V, GUN-4WT and GUN4-V [22], X4 HIV-1 strain IIIB [23] and R5 HIV-1 strain BaL [24] were used. All of these HIV-1 strains were classified as subtypes B [25] based on the amino acid sequences of their envelop proteins. As for HIV-2 strains, CBL23, ROD and SBL6669, and SIV strains, mac251 and mndGB-1, were used. Primary isolates of HIV/SIV were obtained from the National Institute for Biological Standard and Control (NIBSC, London, UK). Their origins, subtypes and NIBSC-reference codes are as follows: HIV-1 strains, MVP-5180 (Cameroon, subtype O, EVA167), 93BR020 (Brazil, subtype F, ARP179.25), 92US723 (USA, subtype B, ARP1039.3), HAN2 (Germany, subtype B, EVA158). An HIV-2 strain, MIR (Guinia Bissau, EVA171), and an SIV primary isolate, smE660 (ARP1040), originated from a sooty mangabey, were also used. Primary isolate, GUN11 was isolated from Gunma University Hospital and included in this study.

### Detection and Amplification of CCR6 Open Reading Frame (ORF)

The reference DNA sequence for the coding region of CCR6 was obtained from the GenBank database. Oligonucleotide primers were designed to cover its ORF and synthesized (Proligo K.K., Tokyo, Japan) to detect reverse-transcribed mRNA by PCR. Names of PCR primers, their sequences, orientations and positions in the respective open reading frames are as follows: CCR6-F, 5´ ATGAGCGGGGAATCAATGAATTTCAGCGAT 3´(sense: from the 1^st^ to the 30^th^ position) and CCR6-R, 5´ TCAGCTTTCTATCACATAGTGAAGGACG 3´(antisense: 1110^th^ -1137^th^) [GenBank accession number, U 60000.1]. As controls, the mRNA expressions of CD4 (M12807.1), CCR5 (U54994.1), CXCR4 (AY242129.1) and gleceraldehyde-3-phospahte dehydrogenase (GAPDH, M17851.1) were also examined. Total RNA was isolated from human cell lines and PBMC using ISOGEN (Nippon Gene, Tokyo, Japan) according to the manufacture’s protocol. Preparation of cDNA followed by amplification of CCR6-ORF has been done by the procedure described earlier [26]. As control, mRNA of GAPDH was amplified by RT-PCR and examined by electrophoresis through 1% (w/v) agarose gel.

### Cloning of CCR6 as Coreceptor Candidate

DNA fragments for the full length ORFs of CCR6 were cloned into a TA-cloning plasmid, pGEM-T Easy (Promega, Madison, WI), and the derivative plasmids were designated as pGEM-T Easy/CCR6. DNA sequencing of TA cloned derivatives was performed by a 5500 DNA sequencer (Hitachi, Tokyo, Japan) using fluorescent primers labeled with Texas Red. Cloned CCR6 ORFs were separated from TA plasmids by restriction-digestion and recloned into an expression plasmid, pMX-puro [27]. Plasmid with correctly oriented CCR6-insert was transfected into NP-2/CD4 cells to produce and establish NP-2/CD4/CCR6 indicator cell lines as described earlier [15,26]. As HIV/SIV-susceptible positive-control cells, NP-2/CD4/CCR5 and NP-2/CD4/CXCR4 were established. NP-2/CD4 and NP-2 cell lines were used as negative-controls.

### HIV/SIV Infection Assay

For viral infection assay, NP-2/CD4/CCR6, NP-2/CD4/CCR5, NP-2/CD4/CXCR4 and NP-2/CD4 cells were seeded into wells of 24-well plates at a density of 5×10^4^ cells/ml. On the following day, medium was taken off and viral inoculums were added to the seeded-cells. After 6 hour incubation, the cells were washed three times with EMEM containing 10% FBS to remove free virus, filled finally with 500 µL fresh medium per well and cultured at 37^°^C in 5% CO_2_ incubator. The cells were passaged every 3-5 days and maintained up to 8 weeks. The expression of HIV/SIV antigens in the infected cells was detected by indirect immunofluorescence assay (IFA) [28]. Viral induced cell fusions were observed under light microscopy. Syncytia-formation was detected by Giemsa staining (Muto Pure Chemicals, Tokyo, Japan). Subsequently, virus-releases through different coreceptors were quantified by measuring reverse transcriptase (RT) activities in culture supernatants of respective cell lines following the gold standard method [16].

### PCR Amplification, Cloning and Sequencing of Viral DNA

We conducted PCR to detect proviral DNA for the Env-coding regions using the genomic DNA of infected cells as templates [29]. For HIV-2MIR, the primer pair used was HIV2-F (forward 5´-ATGTGATAAGCACTATTGGGATGATA-3´) and HIV2-R (reverse 5´-CATGCTTGTTTAGGTCTTGTATTGAT-3´), located between positions of 7084-7109 and 7433-7458, respectively (nucleotide positions of HIV-2CRIK, DQ307022.1). This primer pair amplified the V3 loop of *env* gene along with some of the C2 region. For SIVsmE660, a primer set was designed for the V1, V2, and V3 regions of *env* gene as described elsewhere [30]. Detected DNA bands were cloned into a TA-cloning plasmid, pGEM-T Easy, and inserts were sequenced and blasted in the NCBI nucleotide database (http://blast.ncbi.nlm.nih.gov) to confirm the DNA integrity of the inoculated viruses.

### Phylogenetic Analyses

The amino acid sequences of 20 CKRs was collected from UniProtKB/Swiss-Prot database. Alignments of their sub-regions, such as NTRs, extracellular loop-2 (ECL2s), and ECL-3s were performed using the BioEdit program (version 7) and their phylogenetic trees were constructed with the MEGA (Version 5.1, Molecular Evolutionary Genetics Analysis) for windows.

### Nucleotide Sequence Accession Numbers

The nucleotide sequences used in the manuscript have been submitted to GenBank and can be available under the assigned accessions: HIV-2MIR-R5 C1, JN107567; HIV-2MIR-R5 C2, KF148029; HIV-2MIR-R6 C1, JN107569; HIV-2MIR-R6 C2, KF148030; SIVsmE660-R5 C1, JN107564; SIVsmE660-R5 C2, KF148027; SIVsmE660-R6 C1, JN107566 and SIVsmE660-R6 C2, KF148028.

### Ethics Statement

This study was approved by the ethics committee of the Gunma University Faculty of Medicine and written consent was obtained from enrolled healthy blood donor.

## Results

### Detection of CCR5, CXCR4 and CCR6 Expressions

We detected mRNA expression of CCR6 in various cells including T cell line, hepatic cells and PBMC by RT-PCR using the primers specific for the ORF (Table 1). The outcome shows a good harmony to previous findings that reported of CCR6 expression selectively on Th17 cells and regulatory T cells, memory T cells, B cells and dendritic cells [11,31]. Expression of CCR5 was found in ATL-1K, Jurkat, Daudi and in PBMC. CXCR4 expression was similar to that of CCR5 as well as in C8166. CD4 expression was detected in all T cell lines used here and in PBMC, but not in Daudi, as expected (Table 1). NP-2 cells expressed neither CD4 nor coreceptor candidates, therefore, works as an ideal host for coreceptor-study.

**Table 1:** mRNA expression of coreceptor candidates, CD4 and GAPDH in various cells.

Candidate	Cells expressing mRNA^a^							
	ATL-1K	C8166	Daudi	HepG2	huH1	Jurkat	NP2	PBMC
CCR6	**++**	**++**	**-**	**++**	**+**	**-**	**-**	**++**
CCR5	**++**	**-**	**++**	**-**	**-**	**++**	**-**	**++**
CXCR4	**++**	**++**	**++**	**-**	**-**	**++**	**-**	**++**
CD4	**++**	**++**	**-**	**-**	**-**	**++**	**-**	**++**
GAPDH	**++**	**++**	**++**	**++**	**++**	**++**	**++**	**++**

^a^ Cellular RNA was extracted. Reverse transcribed cDNA was amplified by PCR and examined by agarose gel electrophoresis. Intensities of RT-PCR bands are as: - not detected; +, weak; ++, strong.

### Establishment of NP-2/CD4/coreceptor cells

CCR6-ORF amplified from PBMC was transduced into NP-2/CD4 cells to produce NP-2/CD4/CCR6 cells. Similarly, NP-2/CD4/CCR5 and NP-2/CD4/CXCR4 cells were generated with major two corceptors, CCR5 and CXCR4. The expression of CCR5, CCR6 and CXCR4 were affirmed by detecting their specific mRNAs by RT-PCR in respective cell lines. We further confirmed their expressions in NP-2/CD4/coreceptor sub-lines by flowcytometry (FCM) using anti-CCR5 (2D7, BD Bioscience), anti-CXCR4 (12G5, BD Bioscience) and anti-CCR6 (ab10398) monoclonal antibodies (Mabs) following methods described earlier [32]. The parental cell, NP-2/CD4 did not express any of the coreceptor candidates.

### CCR6 is a coreceptor for HIV/SIV

**Figure 1:**
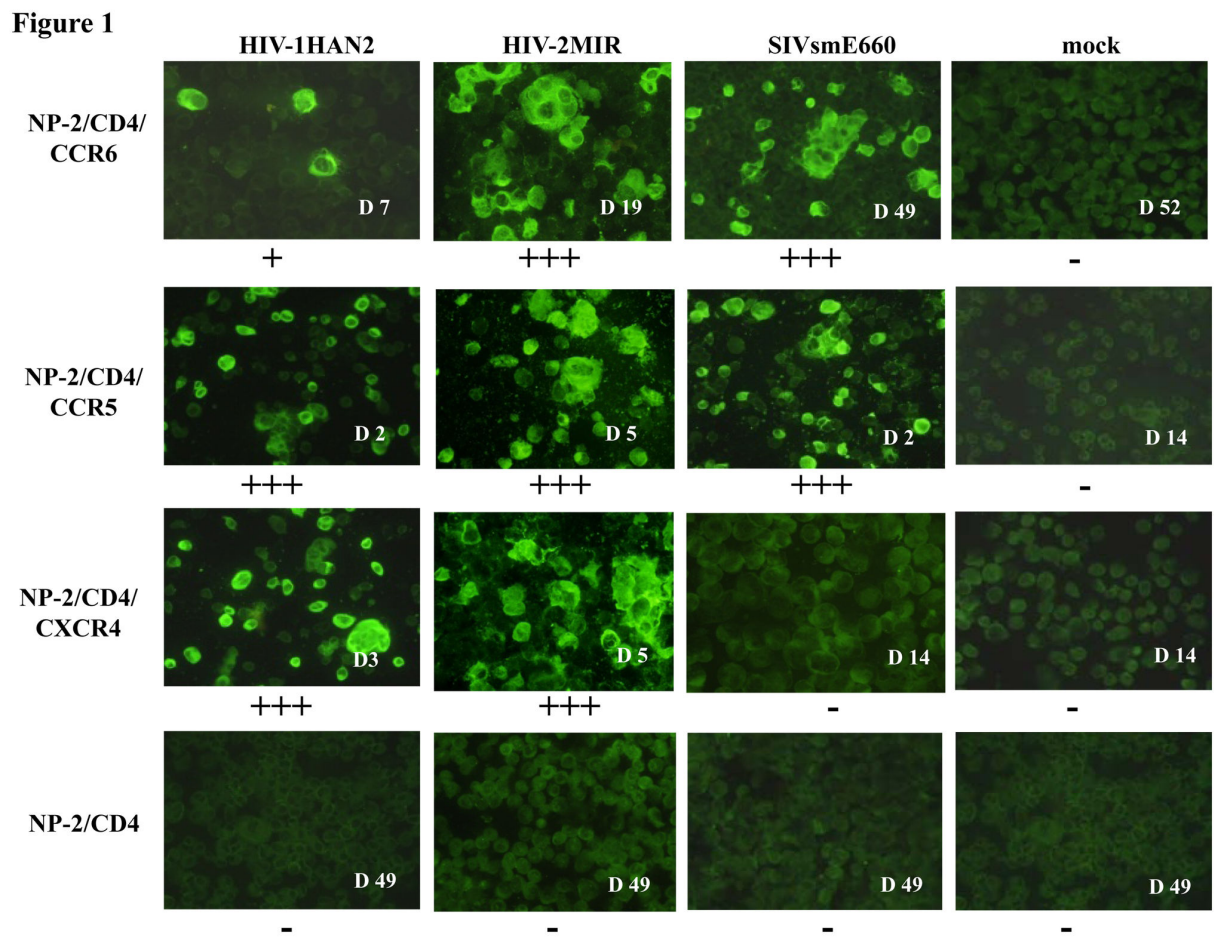
Immunofluorescence assay to detect HIV and SIV infection in NP-2/CD4/GPCR cells. NP-2/CD4/GPCR cells were seeded and next day inoculated with viruses, HIV-1HAN2, HIV-2MIR, and SIVsmE660. Cells were passaged every 3-4 days. Viral antigens in cells were determined by IFA. Briefly, cells were smeared onto glass slides in duplicate, dried, fixed by acetone and stained with pooled human/monkey anti-HIV/SIV sera followed by treatment with fluorescein isothiocynate (FITC)-conjugated goat anti-human/monkey IgG. Days after antigen-appearance of respective virus infection were shown accordingly. Plus (+) and minus (-) signs indicate the level of proviral DNA detection from infected cells.

**Figure 2:**
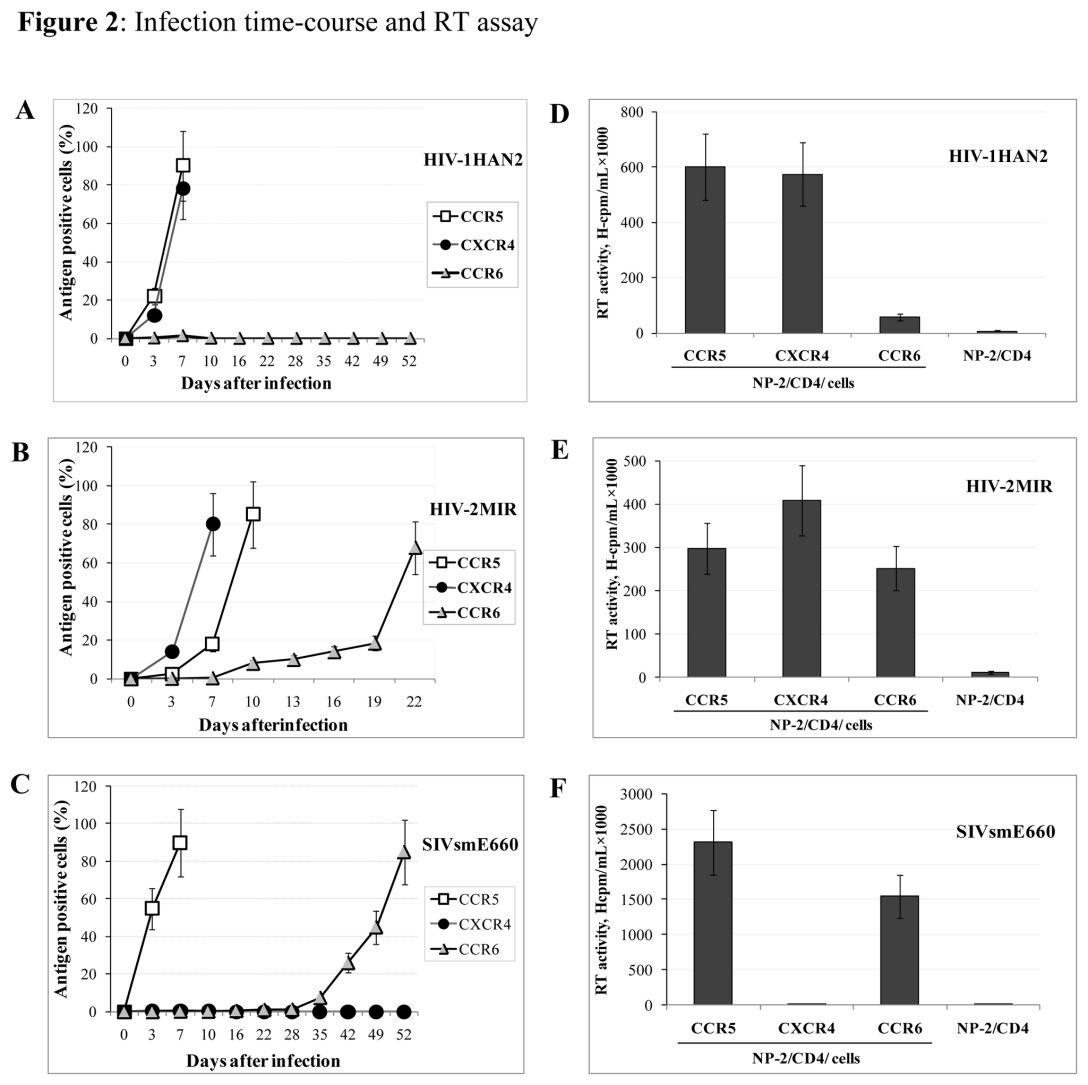
Infection time-course and reverse transcriptase assay of HIV/SIV through different coreceptors. The NP-2/CD4/GPCR cells were seeded and next day inoculated with viruses. Antigen-positive cells were detected by IFA every 3-5 days for up to 7 weeks after inoculation. (**A**) About 2-3% of the NP-2/CD4/CCR6 cells became antigen positive by IFA after seven days of inoculation of HIV-1HAN2. The ratios of HIV-1-positive cells did not increase after this. Most of the cells became infected conveniently using CCR5 and CXCR4 as coreceptors within seven days. (**B**) More than 65% cells became HIV-2MIR antigen-positive three weeks after inoculation using CCR6 as cofactor. Major coreceptors took shorter period to reach similar levels of infection (**C**) NP-2/CD4/CCR6 required seven weeks until most of them became SIVsmE660 antigen-positive. CCR5 took seven days to reach peak levels of infection, whereas CXCR4 did not support infection for SIVsmE660 (**D**) Culture supernatants of HIV-1HAN2-infected cells were harvested for RT activities at day-7 when only 2.5% of NP-2/CD4/CCR6 cells were IFA-positive. Culture media of NP-2/CD4/CCR5 and NP-2/CD4/CXCR4 cells were measured when 80-90% of them became IFA-positive. NP-2/CD4 cells were examined as negative control. The mean cpm of RT activities for the duplicate samples was calculated by subtracting of mean background cpm value. (**E**) Spent culture fluids of HIV-2MIR were measured when NP-2/CD4/CCR6 cells reached to 65% antigen-positive, in comparison to about 85% positives of NP-2/CD4/CCR5 and NP-2/CD4/CXCR4 cells (**F**) RT release of SIVsmE660 was calculated at 80% infection of cells through CCR6 and CCR5 and <0.1% of through CXCR4 as coreceptors.

A coreceptor activity of CCR6 for HIV-1HAN2, HIV-2MIR and SIVsmE660 primary isolates was noticed when viral antigen-positive NP-2/CD4/CCR6 cells were detected by IFA (Figure 1). We detected proviral DNA as alternative evidence in establishing viral infection using CCR6-coreceptor. A few percentages of NP-2/CD4/CCR6 cells became HIV-1HAN2 antigen-positive in 4-7 days after its inoculation (Figure 1): viral antigen-positive cells, however, did not increase during cultivation continued for 7 weeks (Figure 2A). In contrast, HIV-2MIR showed accelerated replication using CCR6 and more than 60% cells became antigen positive within 3 weeks after inoculation (Figure 2B). Similarly, SIVsmE660 started very slow and steady infection through CCR6-coreceptor and about 2% of the cells became IFA-positive after cultivation for up to four weeks. Then the infection progressed rapidly and more than 80% of the cells became SIV antigen-positive seven weeks after infection (Figure 2C). In contrast, all HIV/SIV strains, other than X4 HIV strains IIIB and SBL6669, replicated well in NP-2/CD4/CCR5 cells: most cells became HIV/SIV antigen-positive 3-7 days after inoculation. NP-2/CD4/CXCR4 cells showed high susceptibilities to the viral strains except for HIV-1BaL, SIVmac251, SIVsmE660 and primary HIV-1 isolate 92US723 (Table 2). NP-2/CD4 cells were completely resistant to infection by all HIV and SIV strains tested.

**Table 2:** Replication of HIV-1, HIV-2 and SIV strains and isolates in different CKR-transfected NP-2/CD4 cell lines.

**Virus**		NP-2 cells expressing:			
		CD4, CCR5	CD4, CXCR4	CD4, CCR6	CD4
**Laboratory strains**					
HIV-1	IIIB	-	++++ **^a^**	-	-
	BaL	++++	-	-	-
	GUN1wt	++++	++++	-	-
	GUN1v	++	+++	-	-
	GUN4wt	+++	+++	-	-
	GUN4v	++	+++	-	-
HIV-2	CBL23	++	++++	-	-
	ROD	++	++++	-	-
	SBL6669	-	++++	-	-
SIV	Mac251	++++	-	-	-
	GBmnd1	++++	++	-	-
**Primary isolates**					
HIV-1	MVP5180	++++	++++	-	-
	93BR020	++++	+++	-	-
	92US723	++++	-	-	-
	HAN2	++++	++++	+	-
	GUN11	++++	++++	-	-
HIV-2	MIR	++++	++++	+++	-
SIV	smE660	++++	-	++	-

^a^ Viral antigen positive cells were examined by IFA.

++++,70-90% of cells became antigen-positive at 3-7 days after infection; +++ 70-90% of cells became antigen-positive within 2-3 weeks of infection; ++ 70-90% of cells became antigen-positive within 5-7 weeks of infection; +,1-2% of cells and - < 0.1% of cells became antigen-positive within 7 weeks of infection.

Retroviral cytopathic effects (CPE) in cell-line-system produce large amount of the viruses. The amount of released virus was assessed by reverse transcriptase (RT) activities in spent culture media [33] of viral inoculated cells. For RT assay, culture fluid of infected NP-2/CD4/CCR5 cells at day 7 was collected when 70-90% cells were viral antigen-positive by IFA. However, culture media of NP-2/CD4/CCR6 was examined at day-22 for HIV-2MIR and day-52 for SIVsmE660 when 60-80% respective cells were infected along with CPE. The RT activities of HIV-1HAN2 using CCR5 and CXCR4 as coreceptors were 6.0×10^5^ cpm/mL and 5.8×10^5^ when 80-90% cells become antigen positive by IFA. However, it produced an RT activity of 4.8×10^4^ cpm/mL in replication mediated by CCR6 when only 2.5% of cells were antigen-positive (Figure 2D.). This virus could not increase either antigen-positive cells or RT activity with longer incubation period. On the other hand, HIV-2MIR had produced RT activities of 3.0×10^5^ cpm/mL 4.1×10^5^ cpm/mL at the point of 85-90% infection through CCR5 and CXCR4 respectively. At 70% of infections via CCR6, RT level was 2.5×10^5^ cpm/mL there (Figure 2E). The RT release of SIVsmE660 using CCR5 and CCR6 as coreceptors were 2.3×10^6^ and 1.5×10^6^ cpm/mL, respectively when 80-85% cells become antigen positive (Figure 2F). The virus did not generate RT activity through CXCR4 as shown by IFA. Very negligible RT activities (1.2-1.5×10^3^) were detected when the supernatants of mock-infected cultures were measured.

**Figure 3:**
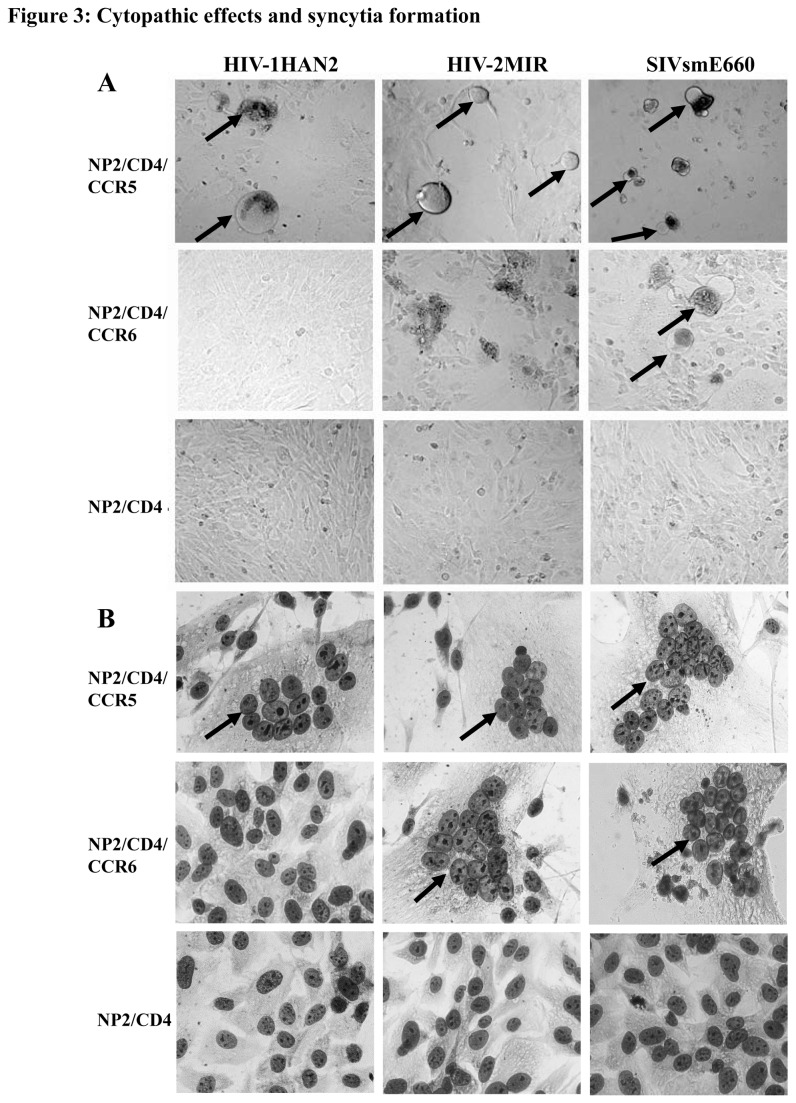
CCR6-induced retroviral cytopathic effects and formation of syncytia. (**A**) Cytopathic effects of HIV/SIV as a sign of acute infection was manifested by ballooning of infected cells and subsequently cell death to release of progeny viruses. Control cells, NP-2/CD4/CCR5 produced ballooning indicated by arrow at four-five days of post infection, however, NP-2/CD4/CCR6 cell generated ballooning with SIVsmE660 virus but not found with HIV-1HAN2 and HIV-2MIR virus infections. (**B**) Viral infected cells were characterized by multinucleated giant cells that were detected by Giemsa staining. NP-2/CD4/CCR5 cells were cultured with viral inoculums for five days and then prepared smear for Giemsa staining. Incubation periods of NP-2/CD4/CCR6 cells for HIV-2MIR and SIVsmE660 were three weeks and seven weeks, respectively to generate MGC. Multinucleated syncytia appeared on infected cells were indicated by arrow. No syncytium was appeared on HIV-1HAN2-exposed NP-2/CD4/CCR6 cells.

With the increase of viral-infected NP-2/CD4/CCR6 cells, ballooning of fused cells were detected under a light microscope as the sign of cytopathic effect [34]. The HIV-2MIR produced more prominent cell-fusions than that of SIVsmE660 through CCR6. HIV-1HAN2 did not produce any ballooning until the end of the assay we conducted (Figure 3A). Majority of HIV and SIV produced CPE as well as syncytia through CCR5/CXCR4-transduced NP-2/CD4 cells. Clusters of multinucleated giant cells (MGC) in syncytia were quantified after fixation and staining with Giemsa. Both HIV-2MIR and SIVsmE660 induced syncytia formation in NP-2/CD4/CCR6 cells, however, HIV-1HAN2 could not form syncytium there (Figure 3B). Clusters were variable in sizes and consisting of 10-20 nucleuses per syncytium. Coreceptor activities of CCR5/CXCR4 frequently generated viral-induced MGC, while coreceptor-negative NP-2/CD4 cells did not form any cluster of nuclei.

### Sequence analyses of the proviral DNAs

**Figure 4:**
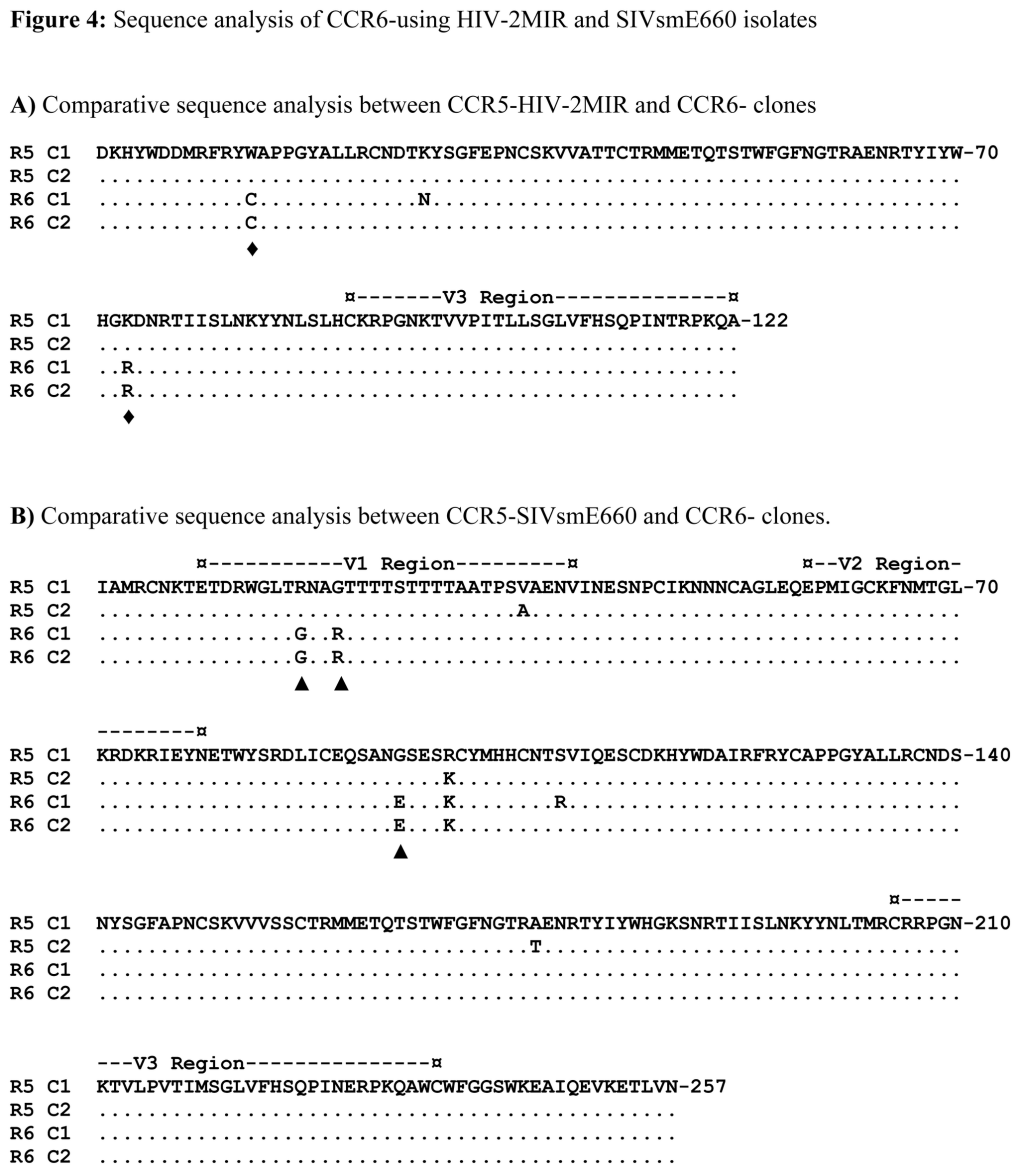
Sequence analysis of CCR6-using HIV-2MIR and SIVsmE660 isolates. The amino acid alignment of the C2-V3 region of the HIV-2MIR-CCR6 variant has been made with parental HIV-2MIR-CCR5 variant. Similarly, genetic divergence of the V1-V3 region of the SIVsmE660-R6 was determined by the pair-wise comparison to its R5-variant. (**A**) Each pair of R6 and R5 variants has been aligned. Dots indicate identity and letters represent substitutions in the adapted variants relative to the parental isolate. The positions of the common amino acid changes were marked by ♦. The V3 domain was indicated by dashes. (**B**) Amino acid sequences of two clones of R6-variant were aligned with two clones of R5-variant in the V1, V2 and V3 domains. The regions equivalent to the V1, V2 and V3 of SIV are indicated by dashes. Dots indicate the identity with the parental isolate; letters represent differences in the adapted variants. The positions of common amino acid changes were marked by ▲.

Due to the high mutation rate of HIV/SIV, the viral populations of CCR5-user may not be similar to those of CCR6-user. We analyzed the genetic divergence of proviral DNAs of HIV-2MIR and SIVsmE660 propagated in NP-2/CD4/CCR5 and NP-2/CD4/CCR6 cells. For HIV-2MIR, we amplified the C2-V3 region of *env* genes and sequenced two clones from each of MIR-R5 and MIR-R6 strains. An overall good integrity was observed in the majority of the nucleotide sequences between R5 and R6 MIR strains. There were four common nucleotide substitutions in the C2 region of MIR-R6 compared to MIR-R5, however, no such changes were found inV3 region (Figure S1). The nucleotide substitutions resulted two amino acid substitutions, namely, tryptophan (W) to cysteine (C) and lysine (K) to arginine (R) in the C2 region common in both clones (Figure 4A). Another change of amino acid from lysine (K) to asparagine (N) was found in one clone of R6-MIR but not in other clone. For SIVsmE660, the *env* gene for the V1-V3 region of R6-variant exhibited six nucleotide substitutions common in two clones relative to that of the parental smE660-R5 ones (Figure S2). Some of the nucleotide substitutions were synonymous that did not result amino acid changes. However, non-synonymous substitutions of nucleotides resulted three amino acid changes such as, arginine (R) to glycine (G) and vice-versa in V1 region, another change from glycine (G) to glutamic acid (E) in C2 region (Figure 4B). Combining, no amino acid substitution were found in the V3 region of either HIV-2MIR or SIVsmE660 while growing through NP-2/CD4/CCR6 cells. In contrast, both of the viruses showed common amino acid substitutions in their C2 regions in using CCR6 as coreceptors.

## Discussion

All HIV/SIV strains or isolates examined in this study were known to use CCR5 and/or CXCR4 as major coreceptors. Many HIV isolates have been found to use FPRL1 [35], CCR2b [36], CCR3 [37], CCR8 [28], CXCR1 [15], CXCR2 [15], CXCR5 [38], CXCR6 [39], CXCR7/RDC1 [40], CX3CR1 [41], D6 [42], APJ [43] and GPR1 [44] as entry co-factors. Most of the reported coreceptors were either CKRs or orphan GPCRs in nature. We did not find detail report of CCR6 to carry out coreceptor function independently so far. Therefore, to examine its novelty among coreceptor family for HIV/SIV is quite inspiring. The expression history of CCR6 in various memory T cells, dendritic cells and Th17 [13] in presence or absence of CD4 and CCR5/CXCR4 has made us more interested to examine its role in HIV infection through this study. Accordingly, the abundant expression of CCR6 in PBMC and the T-cell line C8166, ATL1K was detected, but not in Jurkat cell line (Table 1). We reported the coreceptor role CCR6 for some limited HIV and SIV strains/isolates by various *in vitro* examinations. Another variant of CCR6, known as CKR-L3 [45], was shown to exhibit similar coreceptor function (S. Islam, et al., unpublished data). Active involvement of CCR6 in HIV infection was mentioned in earlier studies where CCR6-expressing Th1 and Th17 cells became more permissive to HIV/SIV infection than that of its CCR6-negative subsets [14,46]. Role of CCR6 was further clarified when ligands to CCR6 was reported to inhibit the viral infection [47].

The function of CCR6 as coreceptor has been revealed by multiple distinct and independent examinations through NP-2/CD4/CCR6 cell-line system. However, NP-2/CD4/CCR6 cells took much longer time to become infected by viruses compared to NP-2/CD4/CCR5 or NP-2/CD4/CXCR4 cells, indicating that CCR6 is much weaker coreceptor than CCR5 or CXCR4. Moreover, CCR6 was found to support infection for three isolates out of 18 HIV/ SIV used in the study and hence justified as a low-efficient entry co-factor than major ones. Further study with high number of various HIV/SIV strains and isolates could clarify our present findings more distinctly. However, CCR6 might be important window for viral infection when alternative coreceptors are blocked or down regulated, as described for alternative coreceptors in CCR5-null situation [8,48].

**Figure 5:**
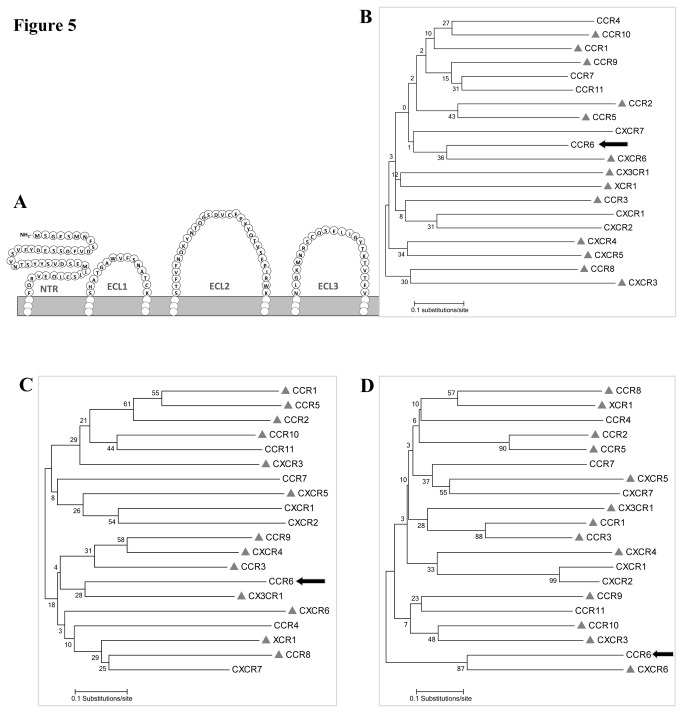
Phylogenetic analyses of coreceptor binding regions of chemokine receptors. (**A**) Schematic diagram was made to present amino terminal region (NTR) and extracellular loops (ECLs) of CCR6 structure. Subsequently three phylogenetic trees were prepared based on NTR, ECL2 and ECL3 of 20 chemokine receptors (CKRs). Branch of CCR6 on each tree was indicated by arrow. CKRs that are reported to function as HIV/SIV coreceptors are marked by “▲”. The phylogenetic tree for NTRs of CKRs (**B**) showed CCR6 homologous to CXCR6. On the other hand, the tree made from ECL2s of all CKRs (**C**) placed CCR6 close to CX3CR1. However, the tree prepared from ECL3s of all CKRs (**D**) positioned CCR6 similar to CXCR6 again.

To find whether a common property is present among the HIV/SIV coreceptors, we examined and analysed the amino acid sequence of NH_2_-terminal region (NTR) of CCR6, CCR5, CXCR4 and other CKRs. Almost all reported HIV/SIV coreceptors possess tyrosine (Y) residues accompanied by aspartic acid (D), asparagine (N) or gluatamic acid (E) residues in their NTRs and tyrosine are reported to play important roles in their coreceptor activities [6,49]. The latest reported coreceptor, N-formylpeptides, FPRL1 that contains two tyrosine residues plus glutamic acids in its NTR, was shown to support the entry of various HIV/SIV strains and isolates [35]. Likely, CCR6, the latest reported coreceptor, also contains three Ys, four Ds and four Es in its NTR (Figure 5A). To discover the relatedness of CCR6-NTR among CKRs, we constructed a phylogenetic tree taking all 20 CKR-NTRs using BioEdit program (Version 7). In the tree, the NTRs were clustered into several distinct branches according to the subfamilies of CKRs. We found CCR6 to form a closely related cluster with CXCR6 (Figure 5B), which was reported HIV- and SIV-coreceptor [39,48], therefore justified its candidacy. Beside NTR, the second or third extracellular loop of coreceptor also play important role in HIV entry [50,51]. Therefore, we prepared two other phylogenetic trees using second and third ECLs of CCR6 to the respective positions of other CKRs. The second ECL of CCR6 placed it close to CX3CR1 (Figure 5C), which is another reported coreceptor [41]. The phylogenetic tree drawn from third ECLs clustered CCR6 again to CXCR6 (Figure 5D), hence reassured its position close to a HIV-coreceptor. Therefore, the hypothesis based on amino acid analyses of NTR and ECLs of CCR6 presented itself a HIV/SIV-coreceptor, as supported by our laboratory investigations.

The third variable region (V3) of HIV-1 gp120 envelope glycoprotein carries essential features for coreceptor binding [52] and determines the types of coreceptor utilization for entry [53]. We deduced the amino acid sequences from the viral DNA sequence of V3 and adjacent *env* regions of HIV-2MIR and SIVsmE660 isolates propagated in NP-2/CD4/CCR6 cells. Upon examination of the V3 and C2 regions of the HIV-2MIR-CCR6 variants in comparison to MIR-CCR5, the V3 region of the CCR6-variants did not show any divergence compared to the parental CCR5-isolate. However, two non-synonymous amino acid substitutions in the C2 region of CCR6-using variant were found. We assume the C2 region could be responsible for the selective use of CCR6 as coreceptor for HIV-2MIR. Similarly, CCR6-using SIVsmE660 exhibited no amino acid substitution in the V3 envelope region. The findings showed good agreement with previous studies describing the region corresponding to the V3 of HIV-1 is conserved in SIV [54] and the genetic variation of SIV is different from HIV-1 [55]. However, this study detected the V1 and V2 as the possible regions in the envelope glycoprotein to exhibiting the genetic variation of SIVsmE660 for selecting CCR6 as new coreceptor and our observation showed harmony to previous reports [56,57].

## Conclusions

Identification of CCR6 as an independent HIV/SIV coreceptor contributed our understanding of virus–host interactions. The expression of CCR6 influences migration of memory CD4+ T-cell subsets into the intestine, brain, and other tissues [58], and may contribute disseminating viral infection. The newly identified coreceptor may promote HIV transmission and disease progression in part, particularly when other major coreceptors are affected. The assumption has been reflected in a recently conducted study in sooty mangabeys that identified the use of alternative coreceptors when CCR5 is mutated [8]. Taking all findings in account, one can presume that the usage spectrum of CKRs or GPCRs as coreceptors by HIV/SIV may be much broader than those of identified to date. However, it remains to be investigated why CCR6 confined its coreceptor-activity limitedly for some HIV/SIV isolates but not for others.

## Supporting Information

Figure S1 **Alignment of the nucleotide sequences of the C2-V3 regions of HIV-2MIR-CCR6 to the parental CCR5-variant.**
(DOCX)

Figure S2 **Alignment of the nucleotide sequences of the V1-V3 regions of SIV-smE660-CCR6 to the parental CCR5-variant.**
(DOCX)
