# Supplementary material for: CCR6 Functions as a New Coreceptor for Limited Primary Human and Simian Immunodeficiency Viruses
Source: PLoS One. 2013 Aug 29;8(8):e73116. doi: 10.1371/journal.pone.0073116 (PMC3757016; doi:10.1371/journal.pone.0073116)
Supplement: Figure S1 — Alignment of the nucleotide sequences of the C2-V3 regions of HIV-2MIR-CCR6 to the parental CCR5-variant. (DOCX) [file pone.0073116.s001.docx]

**Figure S1: Alignment of the nucleotide sequences of the C2-V3 regions of HIV-2MIR-CCR6 to the**

**parental CCR5-variant.**

**10 20 30 40 50 60**

**| | | | | |**

**R5 MIR C1** **GATAAACATTATTGGGATGATATGCGTTTTCGTTATTGGGCGCCGCCGGGCTATGCGCTG-60**

**R5 MIR C2**  **............................................................**

**R6 MIR C1** **......................................C.....................**

**R6 MIR C2**  **......................................C.....................**

**R5 MIR C1** **CTGCGTTGCAACGATACCAAATATAGCGGCTTTGAACCGAACTGCAGCAAAGTGGTGGCG-120**

**R5 MIR C2**  **............................................................**

**R6 MIR C1** **....................C.......................................**

**R6 MIR C2**  **............................................................**

**R5 MIR C1** **ACCACCTGCACCCGTATGATGGAAACCCAGACCAGCACCTGGTTTGGCTTTAACGGCACC-180**

**R5 MIR C2**  **............................................................**

**R6 MIR C1** **............................................................**

**R6 MIR C2**  **............................................................**

**R5 MIR C1** **CGTGCGGAAAACCGTACCTATATTTATTGGCATGGCAAAGATAACCGTACCATTATTAGC-240**

**R5 MIR C2**  **............................................................**

**R6 MIR C1** **....................................CGT.....................**

**R6 MIR C2**  **....................................CGT.....................**

**¤------------------------------**

**R5 MIR C1** **CTGAACAAATATTATAACCTGAGCCTGCATTGCAAACGTCCGGGCAACAAAACCGTGGTG-300**

**R5 MIR C2**  **............................................................**

**R6 MIR C1** **............................................................**

**R6 MIR C2**  **............................................................**

**-----------------------------V3 Region----------------------**

**R5 MIR C1** **CCGATTACCCTGCTGAGCGGCCTGGTGTTTCATAGCCAGCCGATTAACACCCGTCCGAAA-360**

**R5 MIR C2**  **............................................................**

**R6 MIR C1** **............................................................**

**R6 MIR C2**  **............................................................**

**-----¤**

**R5 MIR C1** **CAGGCG-366**

**R5 MIR C2**  **......**

**R6 MIR C1** **......**

**R6 MIR C2**  **......**
