# Supplementary material for: CCR6 Functions as a New Coreceptor for Limited Primary Human and Simian Immunodeficiency Viruses
Source: PLoS One. 2013 Aug 29;8(8):e73116. doi: 10.1371/journal.pone.0073116 (PMC3757016; doi:10.1371/journal.pone.0073116)
Supplement: Figure S2 — Alignment of the nucleotide sequences of the V1-V3 regions of SIV-smE660-CCR6 to the parental CCR5-variant. (DOCX) [file pone.0073116.s002.docx]

**Figure S2: Alignment of the nucleotide sequences of the V1-V3 regions of SIV-smE660-**

**CCR6 to the parental CCR5-variant.**

**10 20 30 40 50 60**

**| | ¤-------------V1 Region-------------**

**R5 smE C1** **ATTGCGATGCGTTGCAACAAAACCGAAACCGATCGTTGGGGCCTGACCCGTAACGCGGGC-60**

**R5 smE C2** **............................................................**

**R6 smE C1** **................................................G.C......C.T**

**R6 smE C2** **................................................G.C......C.T**

**------------------------------------------------------¤**

**R5 smE C1** **ACCACCACCACCAGCACCACCACCACCGCGGCGACCCCGAGCGTGGCGGAAAACGTGATT-120**

**R5 smE C2** **...........................................C................**

**R6 smE C1** **............................................................**

**R6 smE C2** **............................................................**

**¤---------**

**R5 smE C1** **AACGAAAGCAACCCGTGCATTAAAAACAACAACTGCGCGGGCCTGGAACAGGAACCGATG-180**

**R5 smE C2** **............................................................**

**R6 smE C1** **............................................................**

**R6 smE C2** **............................................................**

**----------------------------V2 Region-------------------¤**

**R5 smE C1** **ATTGGCTGCAAATTTAACatgACCGGCCTGAAACGTGATAAACGTATTGAATATAACGAA-240**

**R5 smE C2** **............................................................**

**R6 smE C1** **............................................................**

**R6 smE C2** **............................................................**

**R5 smE C1** **ACCTGGTATAGCCGTGATCTGATTTGCGAACAGAGCGCGAACGGCAGCGAAAGCCGTTGC-300**

**R5 smE C2** **......................................................AAA...**

**R6 smE C1** **...........................................AA.........AAA...**

**R6 smE C2** **...........................................AA.........AAA...**

**R5 smE C1** **TATATGCATCATTGCAACACCAGCGTGATTCAGGAAAGCTGCGATAAACATTATTGGGAT-360**

**R5 smE C2** **............................................................**

**R6 smE C1** **.....................C.T....................................**

**R6 smE C2** **............................................................**

**R5 smE C1** **GCGATTCGTTTTCGTTATTGCGCGCCGCCGGGCTATGCGCTGCTGCGTTGCAACGATAGC-420**

**R5 smE C2** **............................................................**

**R6 smE C1** **............................................................**

**R6 smE C2** **............................................................**

**R5 smE C1** **AACTATAGCGGCTTTGCGCCGAACTGCAGCAAAGTGGTGGTGAGCAGCTGCACCCGTATG-480**

**R5 smE C2** **............................................................**

**R6 smE C1** **............................................................**

**R6 smE C2** **............................................................**

**R5 smE C1** **ATGGAAACCCAGACCAGCACCTGGTTTGGCTTTAACGGCACCCGTGCGGAAAACCGTACC-540**

**R5 smE C2** **.............................................A.C............**

**R6 smE C1** **............................................................**

**R6 smE C2** **............................................................**

**R5 smE C1** **TATATTTATTGGCATGGCAAAAGCAACCGTACCATTATTAGCCTGAACAAATATTATAAC-600**

**R5 smE C2** **............................................................**

**R6 smE C1** **............................................................**

**R6 smE C2** **............................................................**

**¤---------------------------V3 Region-----------**

**R5 smE C1** **CTGACCATGCGTTGCCGTCGTCCGGGCAACAAAACCGTGCTGCCGGTGACCATTATGAGC-660**

**R5 smE C2** **............................................................**

**R6 smE C1** **............................................................**

**R6 smE C2** **............................................................**

**--------------------------------------------------¤**

**R5 smE C1** **GGCCTGGTGTTTCATAGCCAGCCGATTAACGAACGTCCGAAACAGGCGTGGTGCTGGTTT-720**

**R5 smE C2** **............................................................**

**R6 smE C1** **............................................................**

**R6 smE C2** **............................................................**

**R5 smE C1** **GGCGGCAGCTGGAAAGAAGCGATTCAGGAAGTGAAAGAAACCCTGGTGAAC-771**

**R5 smE C2** **...................................................**

**R6 smE C1** **...................................................**

**R6 smE C2** **...................................................**
